# Decreased ADP-Ribosyl Cyclase Activity in Peripheral Blood Mononuclear Cells from Diabetic Patients with Nephropathy

**DOI:** 10.1155/2008/897508

**Published:** 2009-03-17

**Authors:** Michio Ohtsuji, Kunimasa Yagi, Miyuki Shintaku-Kubota, Yukiko Kojima-Koba, Naoko Ito, Masako Sugihara, Naoto Yamaaki, Daisuke Chujo, Atsushi Nohara, Yoshiyu Takeda, Junji Kobayashi, Masakazu Yamagishi, Haruhiro Higashida

**Affiliations:** ^1^Division of Endocrinology and Diabetology, Kanazawa University Graduate School of Medicine, 13-1 Takaramachi, Kanazawa 920 8640, Japan; ^2^Division of Cardiovascular Medicine, Department of Internal Medicine, Kanazawa University Graduate School of Medicine, 13-1 Takaramachi, Kanazawa 920 8640, Japan; ^3^Department of Biophysical Genetics, Kanazawa University Graduate School of Medicine, 13-1 Takaramachi, Kanazawa 920 8640, Japan

## Abstract

*Aims/hypothesis*. ADP-ribosyl-cyclase activity (ADPRCA) of CD38 and other ectoenzymes mainly generate cyclic adenosine 5’diphosphate-(ADP-) ribose (cADPR) as a second messenger in various mammalian cells, including pancreatic beta cells and peripheral blood mononuclear cells (PBMCs). Since PBMCs contribute to the pathogenesis of diabetic nephropathy, ADPRCA of PBMCs could serve as a clinical prognostic marker for diabetic nephropathy. This study aimed to investigate the connection between ADPRCA in PBMCs and diabetic complications. *Methods*. PBMCs from 60 diabetic patients (10 for type 1 and 50 for type 2) and 15 nondiabetic controls were fluorometrically measured for ADPRCA based on the conversion of nicotinamide guanine dinucleotide (NGD^+^) into cyclic GDP-ribose. *Results*. ADPRCA negatively correlated with the level of HbA1c (*P* = .040, *R* ^2^ = .073), although ADPRCA showed no significant correlation with gender, age, BMI, blood pressure, level of fasting plasma glucose and lipid levels, as well as type, duration, or medication of diabetes. Interestingly, patients with nephropathy, but not other complications, presented significantly lower ADPRCA than those without nephropathy (*P* = .0198) and diabetes (*P* = .0332). ANCOVA analysis adjusted for HbA1c showed no significant correlation between ADPRCA and nephropathy. However, logistic regression analyses revealed that determinants for nephropathy were systolic blood pressure and ADPRCA, not HbA1c. *Conclusion/interpretation*. Decreased ADPRCA significantly correlated with diabetic nephropathy. ADPRCA in PBMCs would be an important marker associated with diabetic nephropathy.

## 1. INTRODUCTION

Diabetes mellitus is characterized by chronic hyperglycemia and the development of diabetes-specific microvascular complications. As a consequence of these complications, diabetes is a leading cause of end stage renal disease (ESRD) [1]. Actually, diabetic nephropathy is the single most common cause of ESRD in Japan [2]. Recent epidemiologic data indicated that the number of Japanese type 2 diabetic patients on renal replacement therapy has tripled within less than 15 years, and Japanese type 2 diabetic patients might be particularly predisposed to nephropathy [3, 4]. Therefore, earlier detection of high-risk Japanese subjects for the diabetic nephropathy is quite important for early intervention to prevent ESRD.

Chronic low-grade inflammation and activation of the innate immune system are closely involved in the pathogenesis of diabetes and its microvascular complications [5, 6]. Inflammatory events are central to the pathogenesis of diabetic nephropathy [7–9], and inflammatory cytokines are involved in its development and progression [10]. Macrophages derived from circulating monocytes have been recently focused on as playing central roles in the progression of diabetic nephropathy [11, 12]. Since PBMCs mainly consist of lymphocytes and monocytes, both of which contribute to the progression of diabetic nephropathy, intracellular regulatory signals involved in the activation of PBMCs could serve as a possible therapeutic target and/or clinical prognostic marker for diabetic nephropathy.

Cyclic adenosine 5’diphosphate-ribose (cADPR) plays a second messenger role in a variety of mammalian cells, including pancreatic beta cells, kidney mesangial cells, and PBMCs [13]. The rapid release of Ca^2+^ from the smooth endoplasmic reticulum (SER), the most characterized Ca^2+^ organelle, is evoked by stimulation of two kinds of receptors on the SER for ryanodine and inositol (1,4,5)-triphosphate. cADPR is catalyzed from beta-NAD^+^ by ADP-ribosyl-cyclase activity (ADPRCA) of CD38, CD157, and other ectocellular membrane-bound enzymes [14–18]. cADPR has been recently demonstrated to stimulate a variety of mammalian cell functions, including proliferation, secretion, contraction, and vasodilation [19–25]. For example, several studies have examined ADPRCA in kidney mesangial cells [26, 27], and cADPR has been reported to induce smooth muscle contraction in small renal arteries [28].

Since cADPR modulates adaptive immune recognition process of PBMCs [29], we hypothesized that impaired regulatory signals of ADPRCA in PBMCs would result in the progression of diabetic vascular complications. However, no clinical studies have been performed to examine the relationship between CD38 and diabetic complications.

Based on this background, this study investigates the connection between ADPRCA in PBMCs and diabetic vascular complications, especially nephropathy.

## 2. MATERIALS AND METHODS

### 2.1. Study population

Patients were eligible for the study if they exhibited diabetes mellitus. Diabetes mellitus was diagnosed and classified according to World Health Organization (WHO) criteria [30, 31]. Subjects with other endocrine diseases or significant renal and/or hepatic diseases were excluded. The study, approved by the ethics committee of Kanazawa University Graduate School of Medical Science, was conducted in accordance with the Declaration of Helsinki (1964), and all patients gave written informed consent before participating in the study.

**Definition of diabetic complications**

ssessment and diagnosis of diabetic complications were performed as below [32]. Nephropathy was diagnosed as the existence of albuminuria, proteinuria, and/or creatinine clearance <60 mL/min. Albuminuria was defined as urinary albumin excretion between 20 and 200 mg/24 hr or urinary albumin to urinary creatinine ratio between 30 and 300 mg/gCr. Proteinuria was defined as Albustix [Ames] positive. Creatinine clearance values were calculated by the Cockroft-Gault formula. Retinopathy was diagnosed on the basis of direct ophthalmoscopy (through a dilated pupil) by an experienced ophthalmologist and/or by fluorescein angiography. Peripheral neuropathy was assessed by questioning patients about symptoms of neuropathy, including paresthesia, dulled sensation, and pain in legs and feet and was based on clinical examination (i.e., measuring abnormal knee/ankle reflexes and a Semmes-Weinstein monofilament) and confirmed by measurement of conduction velocities of ulnar (motor and sensory), tibial, peroneal, and sural nerves. Macroangiopathy was considered in subjects who met the following three conditions: (1) a history of a cardiovascular event and/or the presence of angina and/or permanent ischemic electrocardiogram abnormalities at rest or ischemic abnormalities in a stress test (usually combined with a cardiac noninvasive imaging technique); (2) claudication and/or abolished peripheral pulses and/or foot lesions due to vascular disease demonstrated by Doppler echography and/or angiography; or (3) carotid vascular disease, as assessed by Doppler echography.

**Medication for diabetes**

Medication for type 2 diabetics was classified into three categories: dietary therapy without medication, insulin injection, and oral hypoglycemic agents including sulphonylureas and alpha-glucosidase inhibitors. No type 2 diabetic subjects being treated with insulin sensitizers (including thiazolidinediones and biguanides) were enrolled in this study.

### 2.2. Laboratory measurements

Body mass index (BMI) was calculated as weight (in kilograms) divided by height (in meters) squared. Venous blood samples were obtained after a 12-hour overnight fast. Blood glucose was measured with the glucose oxidase method and HbA1c by high-pressure liquid chromatography. Serum total cholesterol (TC) and triglycerides (TG) were determined by enzymatic methods, and high-density lipoprotein cholesterol (HDL-C) levels were measured by a polyanion-polymer/detergent method. Low-density lipoprotein cholesterol (LDL-C) was calculated using the Friedewald formula.

### 2.3. Measurement of ADPRCA

**Isolation of PBMC membranous fraction**

After obtaining written informed consent, peripheral blood samples were collected. PBMCs were isolated using Ficoll-Paque PLUS (Sigma) [33–35] and centrifuged. Isolated PBMCs were suspended in 10 mM Tris/HCl solution, pH 7.4, with 5 mM MgCl_2_ (2 mL for each sample) at 4°C for 30 minutes. The suspension was homogenized in a Teflon-glass homogenizer; the resultant homogenate was centrifuged at 4°C for 5 minutes at 1000×g to remove unbroken cells and nuclei. Crude membrane fractions were prepared by centrifugation (twice) of homogenates at 105 000×g for 15 minutes. The supernatant was removed, and precipitates were suspended in 10 mM Tris/HCl solution, pH 6.7 [36, 37]. For each experiment, membranes were freshly prepared and used immediately for enzymatic reactions.

**Fluorometric measurement of ADP-ribosyl cyclase activity**

ADPRCA was determined fluorometrically using a technique based on measurement of the conversion of *β*-NGD^+^ into the fluorescent product cGDP-ribose [36–39]. In brief, 2.5 mL of reaction mixtures containing 60 *μ*M *β*-NGD^+^, 50 mM Tris/HCl, pH 6.6, 100 mM KCl, 10 *μ*M CaCl_2_, and membranes (62–297 *μ*g of protein) were maintained at 37°C under constant stirring. The samples were then excited at 300 nm, and fluorescence emission was continuously monitored at 410 nm in a Shimadzu RF-5300PC spectrofluorophotometer (Kyoto, Japan). Activity was calculated from the data points recorded during 5 minutes per mg membrane protein as reported previously [37].

### 2.4. Statistical analysis

All data are shown as mean ± SD. Continuous variables were compared by one-way analyses of variance (ANOVA) or covariance (ANCOVA) after being adjusted for age, BMI, and sex. Normality of the distribution of Ln(ADPRCA) was confirmed by Shapiro-Wilk's W-test. Differences between the two groups were compared by chi^2^ analysis (categorical variables) or nonparametric Mann-Whitney U-test (continuous variables). Logistic regression analyses were performed to clarify the clinical parameters contributing to categorical variables. Stepwise multivariate regression analyses were performed to clarify the clinical parameters contributing to the level of continuous variable. All statistical analyses were conducted with JMP 6.03 for Macintosh OS-X 10.5 (SAS Institute Inc, Cary, NC, USA). A *P*-value of less than .05 was considered statistically significant.

## 3. RESULTS

**Relationship between ADPRCA and clinical parameters**

**Figure 1:**
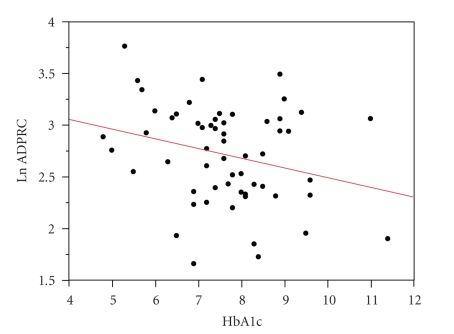
ADP-ribosyl cyclase activities and the level of HbA1c. Statistically significant relationship was observed between ADPRCA and HbA1c (*P* = .040, *R* ^2^ = 0.073, Ln(ADPRC) = 3.4277567–0.093904*HbA1c).

**Figure 2:**
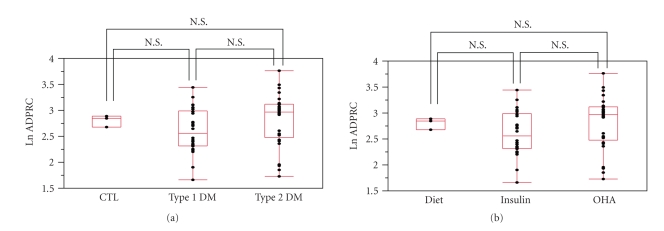
(a) ADP-ribosyl cyclase activities and type of diabetes. No significant relationship was observed between ADPRCA and type of diabetes. (b) ADP-ribosyl cyclase activities and medication for type 2 diabetes. No significant relationship was observed between ADPRCA and medication for type 2 diabetes. CTL: control; OHA: oral hypoglycemic agent.

In total, sixty Japanese diabetic patients (31 males and 29 females) and fifteen nondiabetic controls (6 males and 9 females) were enrolled in this study. The baseline characteristics of the subjects are shown in Table 1. ADPRCA showed statistically significant negative correlation with the level of HbA1c (*P* = .040, *R* ^2^ = 0.073, Figure 1). As shown in Table 2, no significant correlation was observed between ADPRCA and other clinical parameters (level of fasting plasma glucose, systolic and diastolic blood pressure, serum total cholesterol, logarithm of serum TG, HDL-C, LDL-C, age, duration of diabetes, BMI, gender difference, type of diabetes (Figure 2(a)), or medication for diabetes (Figure 2(b))).

**ADPRCA in individuals with diabetic complications**

**Figure 3:**
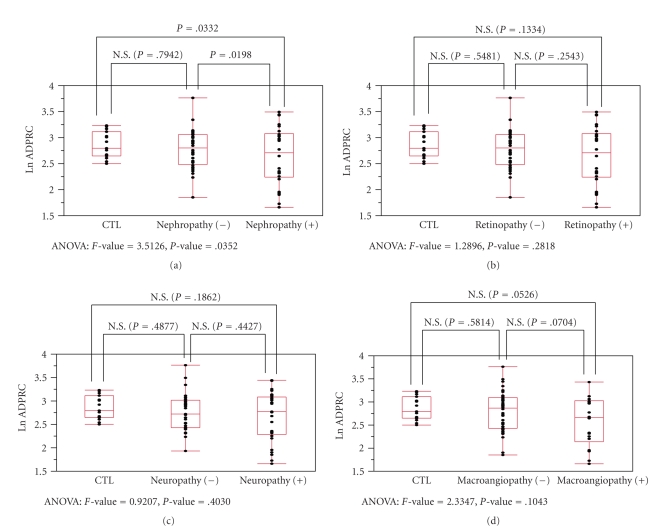
Relationship between logarithm of ADP-ribosyl-cyclase activity Ln(ADPRCA) and diabetic vascular complications. Figures show relationship between nondiabetic control, subjects with each complication, and those without. (a) Subjects with nephropathy showed lower ADPRCA than those without nephropathy (*P* = .0198) and nondiabetic controls (*P* = .0332). (b)–(d) No significant difference in ADPRCA was observed in subjects with retinopathy, neuropathy, and macroangiopathy. CTL: control.

Figure 3 shows the relationship between ADPRCA and the diabetic vascular complications. ADPRCA was significantly lower in subjects with nephropathy than those without (*P* = .0198) and nondiabetic controls (*P* = .0332) (Figure 3(a)). Subjects with other complications also showed similar tendencies; however, no significant correlations between ADPRCA and retinopathy, neuropathy, or macroangiopathy were observed (Figures 3(b)–3(d)). Although HbA1c level showed relationship with ADPRCA, no significant difference was observed in ADPRCA between subjects with any diabetic complications (data not shown).

As HbA1c was significantly related to ADPRCA, we examined whether HbA1c contributed to the relationship between ADPRCA and nephropathy. The results of ANCOVA showed that the difference just failed to meet statistical significance after adjusting for HbA1c (Table 3). Next, to examine whether ADPRCA could be an independent contributor to the existence of nephropathy, we performed logistic analyses adjusted for systolic blood pressure, diastolic blood pressure, TG, HDL, LDL, gender, type of diabetes, duration of diabetes, medication for diabetes, HbA1c, and BMI. Systolic blood pressure and ADPRCA contributed significantly to the existence of nephropathy (Table 4).

## 4. DISCUSSION

The main finding of this study was that diabetic subjects with nephropathy showed decreased ADPRCA. However, PBMCs in proinflammatory states like diabetic vasculopathy might be relating to increased ADPRCA as several cytokines including IL-8, IFN-gamma upregulate intracellular CD38 activity [17], our results interestingly showed decreased ADPRCA in PBMCs. Logistic analysis revealed that only systolic blood pressure and ADPRCA, but not HbA1c, were significantly related to the incidence of nephropathy. Therefore, contribution of HbA1c to the relationship between ADPRCA and nephropathy should be considered small in extent. ADPRCA's correlation with nephropathy seems reasonable.

First, to discuss the role of cADPR-mediated signals in PBMCs, since ADPRCA could be stimulated by angiotensin-II [37], kidney tissue with diabetic nephropathy could show increased ADPRCA. Recent report by Kim et al. showed increased ADPRCA in the kidney of STZ-induced diabetes mice [18]. As we measured ADPRCA in PBMCs, this discrepancy could be acceptable. Interestingly, the roles of CD38 on PBMCs' effect on vascular complications are bidirectional. In PBMCs, binding of agonistic anti-CD38 antibodies, which stimulate ADPRCA, induces release of proinflammatory cytokines including IL-1, IL-6, and TNF-alpha over the short term [40]. Cytokine release could make an important contribution to inflammation responsible in the early stages of diabetic vascular complications. On the other hand, agonistic CD38 ligation inhibits cell growth and induces apoptosis in B-cell precursors [41] mediating phosphatidylinositol 3-kinase signaling [42], although having stimulatory effects on mature lymphocytes. The suppressive effect mediated by CD38 was also observed in experiments with patient-derived myeloid leukemia cells and with the murine cell line [43]. In addition, CD38 expression has been reported in circulating monocytes but not in resident macrophages and dendritic cells [44, 45]. Differentiation of monocytes to macrophages resulted in the downregulation of surface expression of CD38 [46]. CD38 is strongly expressed in lymphocyte precursors, declined during differentiation, and then upregulated again in mature plasma cells [47]. CD157 was suggested to display a similar expression tendency in myeloid cells [48]. Since hyperglycemia directly enhances protein ADP-ribosylation in cultured neuroblastoma cells [49], resulting in increased ADPRCA in diabetic subjects, we speculate that decreased ADPRCA in PBMCs could reflect decreased suppressive effects of CD38 and CD157 and increased numbers of differentiated cells.

Second, let us consider the agonistic effects of autoantibodies against CD38, which is shown to exert insulin secretion from cultured human islets [50] through ADPRCA activation. In humans, the majority of anti-CD38 autoantibodies (~60%) display agonistic properties [51, 52], which demonstrate the capability to trigger Ca^2+^ release in lymphocytic cell lines [53]. In agreement with these functional features, the presence of anti-CD38 autoantibodies in type 2 diabetic patients was associated with significantly higher levels of fasting plasma C-peptide and insulin, as compared with anti-CD38 negative subjects. Thus, anti-CD38 autoimmunity might indicate a relative protection against beta-cell failure and a lower risk of insulin requirement [52, 54, 55]. Previous reports on the clinical characteristics of anti-CD38 autoantibodies carriers have not gone into depth on diabetic complications, although the possible exacerbation of diabetic complications by the agonistic effect of anti-CD38 autoantibodies on PBMCs was noted by Mallone et al. [51]. However, several discussions should be needed for the relationship between CD38 autoantibodies and diabetic vasculopathy as agonistic CD38 autoantibodies possibly stimulate both insulin rsecretion resulting in hyperinsulinemia, the prominent risk factor for diabetic macroangiopathy, and angiotensin-II induced renal artery contraction. We just speculate that those with anti-CD38 autoantibodies should show lower frequencies of diabetic nephropathy, and other vascular complications in long-term follow-up studies due to better glycemic control and increased ADPRCA reflecting less maturated PBMCs.

Third, as all of our subjects were diabetic, it behooves us mention the Okamoto model of diabetes pathogenesis. CD38-related signal has been well documented in diabetes mellitus [18, 56–58]. Our results showing decreased ADPRCA being significantly correlated to increased HbA1c is compatible with previous studies reflecting to some degree the Okamoto model [59–61]. Interestingly, in our study, no significant relationship was observed between ADPRCA and fasting plasma glucose. We suggest that ADPRCA might be more related to postprandial than fasting plasma glucose level, the former being strongly responsive to insulin secretion [27, 29].

Finally, the clinical significance of our results: CD38 expression, representative of ADP-ribosyl cyclase, is already used clinically as a prognostic marker for HIV infected subjects [62, 63] and chronic lymphoid leukemia (CLL) [13, 56, 64]. Since other ADP ribosyl cyclases including CD157 reside on the cell surface of PBMCs, restricting examination to CD38 would not entirely cover cADPR-mediated signaling impairment. Measuring ADPRCA would thus be a better approach in flagging up groups at risk for diabetic nephropathy from among general diabetic subjects. We envision ADPRCA measurement being similarly introduced into clinical settings for detecting subjects at high-risk for diabetic nephropathy and thus prompting early intervention to prevent ESRD.

This study had a number of limitations. First, we did not have data on anti-CD38 autoantibodies. ADPRCA in each subject is partly determined by preexisting factors independent of diabetes, including CD38 genetic polymorphisms and autoantibodies against CD38. We speculate that higher ADPRCA values might reflect possession of anti-CD38 autoantibodies. Second, we did not examine any markers of lymphocyte or monocyte maturation/differentiation. Both should be examined in further studies.

In conclusion, our findings suggest that decreased ADPRCA in PBMCs is significantly correlated with diabetic nephropathy and that measurement of ADPRCA in PBMCs could serve as an important marker associated with diabetic nephropathy. Further prospective studies should be introduced to clarify the mechanism and predictive significance of decreased ADPRCA in diabetic complications.

## Figures and Tables

**Table 1:** Baseline characteristics of subjects. Results are expressed as mean ± S.D.

	Type 1 diabetes	Type 2 diabetes	Nondiabetic control
Number of subjects	10	50	15
Gender (male/female)	5/5	26/24	6/9
Age (years)	36.0 ± 14.2	63.0 ± 12.0	51.0 ± 22.0
BMI (kg/m^2^)	21.4 ± 2.6	22.9 ± 3.9	22.1 ± 2.4
Duration of diabetes (years)	11.0 ± 9.2	16.0 ± 9.7	—
Fasting plasma glucose (mg/dl)	244 ± 130	164 ± 54	81 ± 12
HbA1c (%)	8.0 ± 1.5	7.6 ± 1.3	4.6 ± 0.7
Medication for diabetes			
* *Diet alone	—	4	—
* *Oral hypoglycemic agents	—	30	—
* *Insulin	10	16	—
Diabetic complications			
* *Nephropathy	3	20	
* *Retinopathy	4	22	
* *Neuropathy	4	25	
* *Macroangiopathy	0	18	
ADP-ribosyl cyclase activity (nmol/min/mg protein)	16.9 ± 7.5	16.6 ± 7.6	17.8 ± 4.6

**Table 2:** Correlation of ADP-ribosyl-cyclase activity with subject parameters (calculated with logarithm-transformed TG).

Factor	*R* ^2^	*P*-value
Fasting plasma glucose	0.0120	NS
HbA1c	0.0731	.040
Systolic blood pressure	0.0133	NS
Diastolic blood pressure	0.0301	NS
Total cholesterol (TC)	0.0424	NS
HDL-C	0.0042	NS
LDL-C	0.0258	NS
Triglyceride (TG)	0.0588	NS
Duration of diabetes	0.0150	NS
Age	0.0367	NS
BMI	0.0174	NS
Gender	0.0151	NS

**Table 3:** Comparison of ADPRCA influence on nephropathy, adjusted for HbA1c.

Factor	Average	S.E.	95% C.I.	*P*-value
Nephropathy (−)	2.799	0.076	2.646–2.951	.075
Nephropathy (+)	2.576	0.095	2.386–2.765	

**Table 4:** Logistic analysis between nephropathy and parameters in diabetic subjects. Carrier of complication = 1, noncarrier = 0; medication: insulin = 2, OHA = 1, diet = 0; gender: male = 1, female = 0; type of diabetes: type 2 = 1, type 1 = 0.

Factor	Regression coefficient	*R* ^2^	*P*-value
Systolic blood pressure	−0.09115	6.040	.0140
ADP-ribosyl-cyclase activity	2.61758	3.707	.0229
